# The Inherited and Familial Component of Early-Onset Colorectal Cancer

**DOI:** 10.3390/cells10030710

**Published:** 2021-03-23

**Authors:** Maria Daca Alvarez, Isabel Quintana, Mariona Terradas, Pilar Mur, Francesc Balaguer, Laura Valle

**Affiliations:** 1 Department of Gastroenterology, Hospital Clínic de Barcelona, Institut d’Investigacions Biomèdiques August Pi i Sunyer (IDIBAPS), University of Barcelona, 08036 Barcelona, Spain; daca@clinic.cat; 2 Hereditary Cancer Program, Catalan Institute of Oncology, Oncobell Program, Bellvitge Biomedical Research Institute (IDIBELL), Hospitalet de Llobregat, 08908 Barcelona, Spain; iquintana@idibell.cat (I.Q.); mterradas@idibell.cat (M.T.); pmur@idibell.cat (P.M.); 3 Centro de Investigación Biomédica en Red de Cáncer (CIBERONC), 28029 Madrid, Spain; 4 Centro de Investigación Biomédica en Red de Enfermedades Hepáticas y Digestivas (CIBEREHD), 28029 Madrid, Spain

**Keywords:** hereditary cancer, cancer genetics, colorectal cancer predisposition, cancer syndrome, lynch syndrome, polygenic risk score

## Abstract

Early-onset colorectal cancer (EOCRC), defined as that diagnosed before the age of 50, accounts for 10–12% of all new colorectal cancer (CRC) diagnoses. Epidemiological data indicate that EOCRC incidence is increasing, despite the observed heterogeneity among countries. Although the cause for such increase remains obscure, ≈13% (range: 9–26%) of EOCRC patients carry pathogenic germline variants in known cancer predisposition genes, including 2.5% of patients with germline pathogenic variants in hereditary cancer genes traditionally not associated with CRC predisposition. Approximately 28% of EOCRC patients have family history of the disease. This article recapitulates current evidence on the inherited syndromes that predispose to EOCRC and its familial component. The evidence gathered support that all patients diagnosed with an EOCRC should be referred to a specialized genetic counseling service and offered somatic and germline pancancer multigene panel testing. The identification of a germline pathogenic variant in a known hereditary cancer gene has relevant implications for the clinical management of the patient and his/her relatives, and it may guide surgical and therapeutic decisions. The relative high prevalence of hereditary cancer syndromes and familial component among EOCRC patients supports further research that helps understand the genetic background, either monogenic or polygenic, behind this increasingly common disease.

## 1. Introduction

Early-onset colorectal cancer (EOCRC), defined as that diagnosed before the age of 50, accounts for 10–12% of all new colorectal cancer (CRC) diagnoses [1,2]. Early-onset cancer, together with familial aggregation of cancer and diagnosis of multiple primary tumors, is one of the hallmarks of inherited cancer predisposition. The identification of a hereditary cancer syndrome, i.e., of a germline pathogenic variant in a hereditary cancer gene, has significant implications for the carriers and their families, as it helps risk assessment, directs clinical management, and guides preventive and therapeutic options [3,4].

The prevalence of hereditary cancer syndromes among EOCRC patients is ≈13% (prevalence range in different reports: 9–26%) [5,6,7,8,9,10,11,12]. The variability observed among studies may be due to (i) different patient inclusion criteria, such as different age cut-offs; (ii) different germline testing approaches, which range from the study of a few major CRC genes to the analysis of multigene hereditary cancer panels or whole exomes; (iii) population-specific founder effects; (iv) variant classification disparities. Table 1 shows the results of different studies assessing the hereditary component of EOCRC. In addition to the known hereditary cancer syndromes—including CRC predisposition syndromes and other cancer syndromes not traditionally associated with CRC—a relevant, still-to-be-determined proportion of EOCRC may be explained by the accumulation of low-risk CRC alleles [13].

In this article we review the inherited cancer syndromes associated with CRC predisposition and their prevalence among EOCRC patients according to the published data, the role of low-risk genetic variants, and the unexplained familial component of EOCRC, finishing with the recommendations for genetic testing and counseling in EOCRC.

## 2. Inherited Syndromes That Predispose to EOCRC

**Figure 1:**
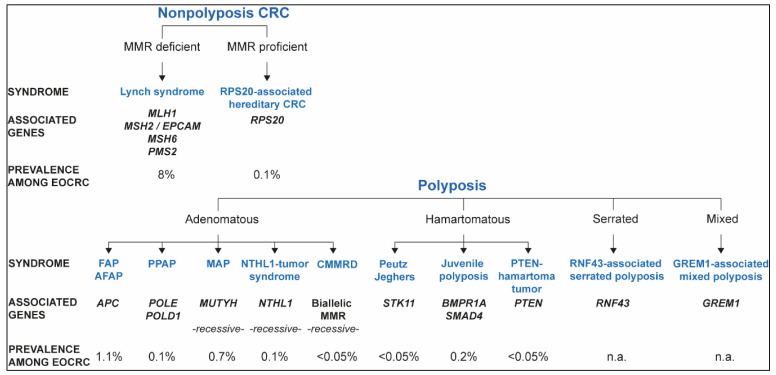
Hereditary colorectal cancer (CRC) syndromes, associated phenotypes, causal genes, and contribution to EOCRC.

Known hereditary colorectal cancer syndromes and their contribution to EOCRC, based on the data recapitulated in this review, are represented in Figure 1. 

### 2.1. Lynch Syndrome and Constitutional Mismatch Repair Deficiency

#### 2.1.1. Lynch Syndrome 

Lynch syndrome is the most common form of hereditary CRC, accounting for 1–3% of all CRC cases [14]. It is an autosomal dominant disorder caused by the presence of a germline pathogenic variant in one of the alleles of the mismatch repair genes *MLHI, MSH2, MSH6*, or PMS2, by a 3′ deletion in the *EPCAM* gene that causes the epigenetic silencing of *MSH2*, or by the constitutional methylation of the *MLH1* promoter [15]. The somatic inactivation of the wildtype allele of the corresponding MMR gene leads to abnormal DNA repair function which causes the accumulation of errors during DNA replication, especially in the repetitive sequences known as microsatellites. As a result, tumors of patients with Lynch syndrome characteristically show MMR deficiency, defined as the presence of microsatellite instability (MSI) and/or loss of MMR protein expression [16]. Individuals with Lynch syndrome have an increased lifetime risk of developing CRC and neoplasms in other organs, including endometrium, ovaries, stomach, small bowel, urinary tract (urothelial), biliary tract, prostate, brain (usually glioblastoma), skin (sebaceous adenomas, sebaceous carcinomas, and keratoacanthomas), and pancreas [14,17]. CRC develops through an accelerated adenoma-carcinoma sequence, and at a younger age compared to sporadic CRC [18]. Lynch syndrome patients have a risk of developing CRC by age 70 of 40–50% for *MLH1* and *MSH2* heterozygous carriers, 20% for *MSH6* carriers and a significant lower risk for PMS2 carriers [14], according to the most recent findings of the international, multicenter Prospective Lynch Syndrome Database calculations (visit www.plsd.eu (accessed on January 2021) for cancer type, gene, and gender-specific risks). In the case of endometrial cancer, the risk by age 70 is 35–46% for *MLH1*, *MSH2* and *MSH6*, and 13% for *PMS2*. The estimated risks largely vary among studies due to their prospective/retrospective nature, ascertainment biases, population analyzed, and statistical methods used [19,20,21,22,23].

Although MMR deficiency is the hallmark of Lynch syndrome, 10–15% of MMR-deficient CRCs are not Lynch syndrome, being this deficiency caused by (i) somatic methylation of the *MLH1* promoter, usually associated with *BRAF* somatic mutations; or (ii) double somatic mutations in the MMR genes, which explains the majority of Lynch-like cases [24], i.e., patients with an MMR deficient tumor with no identified germline pathogenic variant in an MMR gene and no somatic *MLH1* promoter methylation [25]. In line with their most likely sporadic nature, patients with double somatic MMR mutations are diagnosed with cancer at more advanced ages than Lynch syndrome patients, and display less frequently family history of Lynch syndrome tumors. In a small proportion of these cases, double somatic mutations are associated with germline pathogenic variants in other hereditary cancer DNA repair genes such as *MUTYH*, *POLE* or *POLD1*; i.e., due to the germline defect that alters DNA repair or polymerase proofreading and causes accumulation of mutations in the tumor, these may occur in MMR repair genes [26,27,28]. In the Ohio Colorectal Cancer Prevention Initiative, where 450 population-based EOCRC patients were studied, 2% of the CRCs analyzed (9/450), constituting 18.8% of all MMR-deficient EOCRCs, had double somatic mutations in the MMR genes [5]. The same group, by studying a total of 283 MMR-deficient, non-*MLH1* methylated CRC patients not selected by age (populations: USA and Iceland), found that 55% had Lynch syndrome and 32.5% had double somatic MMR gene mutations [24]. Mismatch repair deficiency, whether caused by germline or somatic genetic or epigenetic alterations, leaves a particular mutation pattern in the tumors represented by specific mutational signatures of the Catalogue of Somatic Mutations in Cancer (COSMIC), such as SBS6 [29,30].

The prevalence of Lynch syndrome among EOCRC patients is ≈8% (range: 5–18%) (Table 1), being the distribution among the different MMR genes highly dependent on the population/ethnicity and its relative frequency of founder pathogenic variants [5,6,7,8,9,10,31,32].

The diagnosis of Lynch syndrome is a challenge due to the absence of pathognomonic clinical signs. The presence of a germline pathogenic variant is required to establish the definitive diagnosis. Current diagnostic strategies include the study of families or individuals with familial aggregation and/or early onset of Lynch syndrome-associated tumors; the so-called universal MMR deficiency testing, which implies that all patients with CRC undergo tumor IHC of MMR proteins and/or MSI testing. MMR-deficient cases (excluding those with somatic *MLH1* promoter methylation) should be tested for germline mutations. However, current guidelines (NCCN) recommend performing germline panel testing in all EOCRC. In addition to germline testing, somatic panel testing is also recommended to identify double somatic MMR gene mutations.

#### 2.1.2. Constitutional Mismatch Repair Deficiency (CMMRD)

CMMRD is an autosomal recessive condition associated with a high risk of cancer in children, adolescents and young adults that results from biallelic germline pathogenic variants in the MMR genes (*MLH1, MSH2, MSH6,* or *PMS2)*. MMR deficiency in normal tissues is the hallmark of the syndrome. CMMRD-affected individuals are at high risk of developing hematologic, brain and colorectal cancers, among other tumors, at a remarkably early age. Most patients with CMMRD have non-neoplastic features, with multiple café-au-lait maculae (CALM) being the most prevalent, and also including colorectal and duodenal adenomas. Diagnostic criteria for the clinical suspicion of CMMRD were defined by the European Care for CMMRD Consortium (C4CMMRD) [33]. The CMMRD clinical phenotype includes hematological neoplasms (mainly non-Hodgkin’s lymphoma, lymphoid leukemia, acute myeloid leukemia); brain and central nervous system tumors (more frequently high-grade gliomas, sPNET-supratentorial primitive neuroectodermal tumors), and Lynch syndrome-associated cancers such CRC, small bowel and endometrial cancer.

CRC is diagnosed at very early ages (mean age at diagnosis: 16 years) and current guidelines recommend colonoscopy screening starting at 8 years of age, although adenomatous polyps have been detected even at earlier ages [33,34]. None of the genetic studies performed in ≤55 EOCRC patients shown in Table 1 identified biallelic pathogenic variants in the MMR genes (total number of ≤55 CRC patients where MMR genes were analyzed: 6359), suggesting that the prevalence of CMMRD among EOCRC is extremely low, and/or that the <50 or ≤55 age cutoff defined as inclusion criterium is too high to select for CMMRD cases. In fact, Mork et al. (2015) identified two CMMRD cases among 193 CRC patients diagnosed at age ≤35 years (prevalence 1%) [8], suggesting that the lower the age of CRC diagnosis, the higher the chances of identifying a CMMRD patient.

### 2.2. Nonpolyposis Mismatch Repair Proficient EOCRC: RPS20 and Other Candidate Genes

A large proportion of nonpolyposis familial and/or EOCRCs is not explained by germline alterations in the MMR genes, being their tumors MMR proficient. For years, researchers unsuccessfully looked for their genetic cause. The rapid development of sequencing-based techniques and genome-wide copy number techniques brought hope for the identification of new causal genes for nonpolyposis familial and EOCRC. However, despite the enormous efforts made, which led to the identification of over a hundred candidate genes (reviewed by Terradas et al. [35]), up to date only *RPS20* has shown consistent association with hereditary nonpolyposis CRC. Among the other ≈100 candidate genes identified, nine currently show promising evidence to support their involvement in CRC predisposition: *MRE11*, *BARD1*, *POT1*, *BUB1B*, *POLE2*, *BRF1*, *IL12RB1,* and *PTPN12*, and the epigenetic alteration of *PTPRJ* [35]. Additional candidate genes are published on a regular basis that require validation in large cohorts and functional studies that support their involvement in colorectal carcinogenesis.

*RPS20* was first identified as a hereditary CRC gene in 2014 by Nieminen et al. [36]. *RPS20* c.147dupA (p.Val50Serfs*23) was found in eight CRC-affected members (ages at first CRC diagnosis: 24–75) of a Finnish four-generation family. Six of the eight affected carriers or obligate carriers had developed the first CRC before 55 years of age (24, 33, 43, 50, 52 and 54 years), and three of them had developed metachronous CRCs at age ≤60 years. All studied tumors were MMR proficient and although they did not show loss of the *RPS20* wildtype allele, patients carrying the *RPS20* c.147dupA variant showed a marked increase of 21S pre-rRNAs, supporting a late pre-rRNA processing defect consistent with haploinsufficiency; i.e., the two wild type copies of the gene are required for a normal phenotype. Subsequently, exome sequencing analyses performed on 863 EOCRC patients led to the identification of a disruptive *RPS20* variant (p.Leu61Glufs*11) in a 39 year-old individual with metachronous CRC, and a predicted pathogenic missense variant (p.Val154Leu) in a patient diagnosed with CRC at age 41. In contrast, no rare missense or disruptive *RPS20* variants were found among 1604 cancer-free controls [37]. While our group found no predicted pathogenic *RPS20* variants among 473 familial/EOCRC cases [38], a recent study identified a splicing variant, c.177 + 1G>A, in a family with four CRC-affected members (ages at diagnosis: 38–61), all of them carriers or obligate carriers of the *RPS20* variant [39]. Although information from additional carriers are required to estimate risks and recommend gene-specific surveillance measures, available data suggests low prevalence (allele frequency in familial/early onset CRC patients: 2/2724; 0.07%) and high penetrance (13/16 (81%) >35-year-old carriers of disruptive or canonical splice-site variants were affected with CRC) for *RSP20* pathogenic variants, as well as absence of extracolonic manifestations [35]. 

### 2.3. APC-Associated Polyposis

Familial Adenomatous Polyposis (FAP) is an autosomal dominant syndrome responsible for less than 1% of all CRC cases [40]. It is characterized by the development of hundreds to thousands of adenomas in the colorectum, with a high risk of CRC at an early age. Extracolonic manifestations (duodenal polyps and cancer, desmoid tumor, osteomas, epidermoid cysts, papillary thyroid carcinoma, pancreatic carcinoma, gastric cancer, hepatobiliary, and CNS tumors) are also part of the phenotype [41].

FAP is caused by germline pathogenic variants in the *APC* gene. De novo *APC* mutations are responsible for approximately 25% of FAP cases. Moreover, approximately 20% of individuals with an apparent de novo *APC* mutation have somatic mosaicism (when two or more cell lines in the same individual differ genetically) [42].

FAP may be classified in two categories: (i) the classic form, that consists of the involvement of the entire colon with the development of >100 adenomas (sometimes >1000) during the second decade of life, with a 100% risk of CRC before age 40 years if untreated; (ii) the attenuated form, with 10–100 colorectal adenomas, usually diagnosed later in life and associated with a lower CRC risk (79–80% if untreated) [43]. 

FAP is the second most frequent hereditary cancer syndrome in EOCRC, being the prevalence among EOCRC (age <50 or ≤55) patients 1.1% (range: 0.5–3.3%) (Table 1). Mork et al. [8] detected *APC* pathogenic variants in 6.7% (13/63) of patients diagnosed with CRC at age ≤35, suggesting that the earlier the age at CRC diagnosis, the larger the probability of identifying carriers of *APC* pathogenic variants.

### 2.4. Polymerase Proofreading-Associated Polyposis (PPAP)

Germline pathogenic variants affecting the proofreading activity of polymerases ε (*POLE*) and δ (*POLD1*) cause an autosomal dominant cancer and polyposis predisposing syndrome called polymerase proofreading-associated polyposis (PPAP) [44]. The phenotypic spectrum of PPAP mostly includes colorectal adenomas and CRC, diagnosed in adulthood (mean age 35–50), followed by endometrial, ovarian and breast cancer. Other tumors associated with this syndrome are adenomas and malignant tumors of the upper gastrointestinal tract, brain tumors, cancer of the small intestine and pancreatic cancer [45]. Tumors are diagnosed in the adulthood, except for the rare cases that present a CMMRD-like phenotype, characterized by severe phenotypes, including cancer, in childhood or adolescence [46,47].

The major function of polymerases ε and δ is to replicate the genome, however, unlike other polymerases, ε and δ contain an active 3’-5’ exonuclease domain which proofreads newly synthesized DNA for replication errors. This proofreading is essential for replication fidelity, therefore, its disruption by pathogenic mutations leads to the accumulation of thousands of mutations in the tumors, often >100 mutations per Mb, which is called ultramutated phenotype. Moreover, they present a characteristic spectrum of nucleotide changes that largely differs from that observed in microsatellite stable (MSS) and MSI tumors. This mutation pattern consists of C > A transversions in the context of TCT and C > T transitions in the context of TCG, corresponding to the COSMIC tumor mutational signature SBS10 [29,30]. Heterozygous exonuclease domain mutations, causing 50% of proofreading activity, are enough to increase the mutation frequency, thus not requiring a second hit in the target tissue [44]. Most proofreading-defective tumors are MSS, nevertheless, MSI or somatic DNA MMR gene mutations have been detected in some instances [45], most probably produced by the deficient proofreading activity due to a secondary mutation in an MMR gene. Proofreading deficiency has been associated with good prognosis and excellent response to immune checkpoint inhibition [48].

We recently assessed the prevalence of PPAP among familial and patients by analyzing 2309 prospectively recruited patients assessed in a hereditary cancer clinical program. Our results indicated that PPAP constitutes 0.1–0.4% of familial cancer cases, reaching 0.3–0.7% when only familial/early-onset CRC and polyposis patients were considered [49]. *POLE* and *POLD1* analyses were only covered by Stoffel et al., Laduca et al. and Chubb et al., identifying pathogenic variants affecting the proofreading activity of the polymerases in 0.1% (4/3687) of EOCRC patients (Table 1). 

### 2.5. MUTYH-Associated Polyposis (MAP)

MAP is an autosomal recessive syndrome caused by biallelic pathogenic variants in the *MUTYH* gene. This gene is a component of the base excision repair system that protects genomic information from oxidative damage. Two common founder missense pathogenic variants, p.Tyr179Cys (rs34612342) and p.Gly396Asp (rs36053993), are present in up to 70% of Caucasian patients with this condition and have a carrier frequency of 0.75% in populations of (non-Finnish) European ancestry [50].

Individuals with MAP can exhibit a wide range of phenotypes including classic and attenuated adenomatous polyposis, seldom including cases where no polyps were identified at the time of the CRC diagnosis [51]. MAP-associated tumors and polyps are usually MMR proficient, however, MSI may also be detected due to somatic inactivation of the MMR genes, thus explaining some Lynch-like cases [27,52]. As occurs for other genes involved in DNA repair, a specific mutational signature has been associated with *MUTYH* deficiency, COSMIC signature SBS36, characterized by enrichment of C>A transversions in an NCA or NCT context [30,53,54].

Biallelic *MUTYH* pathogenic variants occur in 0.7% (range: 0.4–2.2%) of EOCRC patients (Table 1).

Several studies indicate that *MUTYH* monoallelic carriers are at slight increased CRC risk (1.5–2.5-fold relative to the general population), particularly in the presence of familial CRC history [55,56], although the evidence is still conflicting. *MUTYH* heterozygotes, who do not develop polyposis, would benefit from population screening measures but could also be offered average moderate-risk colorectal screening based on their family history [57].

### 2.6. NTHL1 Tumor Syndrome

Weren et al. described in 2015 the second recessive inheritable form of adenomatous polyposis caused by biallelic pathogenic variants in a DNA base excision repair gene, affecting, in this case, the Nth like DNA glycosylase 1 (*NTHL1*) [58]. Previously called *NTHL1*-associated polyposis, today, the preferred name for this syndrome is *NTHL1* Tumor Syndrome, based on the high risk to different benign and malignant tumor types that biallelic variants in *NTHL1* confer [59].

To date, 36 individuals from 23 different families carrying biallelic germline pathogenic variants in *NTHL1* have been reported in specific publications [37,58,60,61,62,63,64,65,66]. Unlike other cancer predisposing syndromes, all variants reported in *NTHL1* biallelic carriers are stop-gain, frameshift or canonical splice-site variants; no missense variants have been yet identified as cause of the disease (data obtained October 2020). Over 90% (21/23) of the reported *NTHL1* biallelic carrier families carry the recurrent c.268C>T (p.Gln90Ter) stop-gain variant, being 60% of all (14/23), homozygous carriers.

Regarding the phenotypic expression of the syndrome, all 31 biallelic individuals that underwent a colonoscopy were found to have developed adenomatous polyps (range: 1 > 150) [37,58,60,61,62,63,64,65,66]. Four individuals have developed less than five polyps at ages ranging from 33 to 53, and >10 colorectal adenomas were found in 21/27 (77.8%) patients for which the exact number of polyps was provided. Up to the date of reporting, 20 of the 36 *NTHL1* biallelic carriers (56%) were diagnosed with colorectal cancer, eight of whom diagnosed before the age of 50 years, and the median age at first CRC diagnosis was 53 years (age range: 33–73 years). Due to the ascertainment bias in the reported individuals, accurate cancer risk estimation, mostly for colorectal cancer, has not been possible to calculate; even though, available data suggest that the lifetime risk of CRC in the *NTHL1* tumor syndrome is likely high.

Two thirds (24/36, 66.7%) of all reported biallelic carriers have developed extracolonic malignancies including 15 different tumor types, being breast cancer the most common extracolonic cancer (53% of female carriers were affected with breast cancer), followed by endometrial cancer. Other extracolonic malignancies identified include duodenal polyps and cancer, meningiomas, hematologic malignancies, squamous cell carcinoma, bladder cancer, basal cell carcinoma, cervical cancer, pancreatic cancer, prostate cancer, thyroid cancer, kidney cancer, and brain cancer. Among the 36 *NTHL1* biallelic carriers, 29 (80.6%) were diagnosed with CRC and/or another type of cancer at a median age of 47 years (range: 24–67) at first diagnosis and 18 of them (62.1%) showed an early age of onset (<50 years).

The prevalence of *NTHL1* biallelic germline carriers is rare and has been estimated to be around 1:114,770, five times lower than the estimated prevalence for *MUTYH*-associated polyposis (1:19,079) [67]. The prevalence of biallelic cases among polyposis patients has been estimated to be ≈2% [49,68]. Chubb et al. identified germline biallelic inactivation of *NTHL1* in 0.1% of the EOCRC included in the study. No other studies listed in Table 1 included *NTHL1* in their analyses.

Tumors developed by *NTHL1* biallelic carriers, independently of the tissue of origin, accumulate specific somatic mutations characterized by an enrichment of C > T transitions at non-CpG sites, which translates into a distinct mutational signature, COSMIC signature SBS30 [66,69].

### 2.7. Hamartomatous Polyposis

#### 2.7.1. Peutz–Jeghers Syndrome (PJS)

PJS is a rare autosomal dominant syndrome characterized by pigmentation of mucocutaneous melanin and multiple gastrointestinal polyposis. These polyps, with characteristic histological features (frond-like elongated epithelial component along with cystic gland dilatation and smooth muscle arborization), have a high risk of developing gastrointestinal cancers of the colorectal, pancreatic and gastric organs. In addition, PJS patients have an increased risk for a wide variety of non-gastrointestinal epithelial malignancies, such as cancers of the breast, uterus, cervix, ovary, testicles, and lung. A meta-analysis performed by Hearle et al. and aggregating 419 patients with PJS estimated a cumulative risk for any cancer by age 50 years of 30% and by age 70 of 85%, and a risk for gastrointestinal cancer of 15% and 57% at the respective ages [70]. Mucocutaneous pigmented lesions are seen in around 95% of patients and may be the first clue to an individual having PJS. Such lesions arise in the infancy, occurring around the mouth, but may be seen at other sites such as nostrils, perianal area, fingers and toes, etc. [71].

Germline mutations in *STK11* (also called *LKB1*) are the only known cause of PJS. Between 80% and 94% of families with a PJS phenotype have an identifiable *LKB1*/*STK11* mutation [72]. A clinical diagnosis of PJS can be made when an individual has two or more of the following features: two or more Peutz–Jeghers polyps of the small intestine; typical mucocutaneous hyperpigmentation; and a family history of PJS [73]. The prevalence PJS in the EOCRC population is expected to be low (0.03%, Table 1), and clinical manifestations of the syndrome should guide suspicion (i.e., intussusception or intestinal obstruction due to large polyps in the childhood or adulthood, mucocutaneous hyperpigmentation, etc.). 

#### 2.7.2. Juvenile Polyposis

Juvenile Polyposis (JP) is a rare autosomal dominant syndrome characterized by the presence of juvenile hamartomatous polyps in the gastrointestinal tract, with a lifetime risk of CRC of 20% [74]. JPS is caused by germline pathogenic variants in *SMAD4* or *BMPR1A*, with a mutation in either one identified in 39% of patients with the clinical phenotype. Clinical diagnosis of JP includes the presence of more than five juvenile polyps in the colon and/or rectum; the presence of juvenile polyps along the digestive tract, including the stomach; or the presence of any number of juvenile polyps in association with a family history of JP [75,76].

Congenital defects occur in approximately 15% of JPS cases. A subset of patients with JPS also has hereditary hemorrhagic telangiectasia (HHT), more commonly seen in *SMAD4* mutation carriers and characterized by mucocutaneous telangiectasias, gastrointestinal arteriovenous malformations and pulmonary arterio-venous malformations.

Germline pathogenic variants in either *BMPR1A* or *SMAD4* are identified in 0.2% of EOCRC patients (Table 1).

#### 2.7.3. PTEN Hamartoma Tumor Syndrome

*PTEN* hamartoma tumor syndrome (PHTS), comprising Cowden, Bannayan–Riley–Ruvalcaba, and Proteus-like syndromes, is a rare multisystem disorder associated with increased lifetime risks for several cancer types due to pathogenic germline variants in the tumor suppressor gene *PTEN*. In addition to the increased cancer risk, PHTS is associated with developmental delay, overgrowth phenotypes, including macrocephaly, benign tumors, and skin abnormalities [76]. The most frequent phenotype is called Cowden Syndrome, characterized by multiple hamartomas that can occur in any organ, macrocephaly, mucocutaneous lesions, and an increased risk of several tumors, including CRC. The estimated lifetime risk of cancer in individuals with PHTS range from 85% to 89% for any cancer, 67–85% for female breast cancer, 6–38% for thyroid cancer, 2–28% for endometrial cancer, 2–34% for renal cancer, 9–20% for colorectal cancer, and 0–6% for melanoma [77]. Patients usually present with colorectal polyps (typically hamartomas, but other types such as ganglioneuromas, hyperplastic, adenomatous and inflammatory polyps often occur), and CRC (age at diagnosis: 44–60 years) [78]. Accordingly, the prevalence of germline *PTEN* pathogenic variants among EOCRC patients is low (0.03%), almost negligible (Table 1).

### 2.8. RNF43-Associated Serrated Polyposis

Serrated polyposis syndrome (SPS) is characterized by the presence of numerous colorectal serrated polyps (SP), and it is associated with an increased personal and familial risk of CRC [79,80,81,82]. Although SPS is the most common polyposis syndrome [83,84], its etiology is the least understood. Overall, SPS behaves as a complex disorder consequence of the interaction of both genetic and environmental factors. Despite this complexity, the World Health Organization (WHO) has established specific clinical criteria, recently updated, to standardize the diagnosis and treatment in the clinical practice, as well as to prompt research in the field [85].

Until 2014, previous efforts to identify an inherited genetic cause for SPS had been unsuccessful. That year, Gala et al. provided the first evidence indicating that germline *RNF43* pathogenic variants were associated with SPS [86]. Since then, three truncating and two missense germline pathogenic variants have been reported in 13 carriers from seven families [86,87,88,89,90]. In ClinVar, six different disruptive (stop-gain or frameshift variants) are listed in *RNF43* (date of consultation: Oct 2020). Of the seven reported carrier families, five carry loss of function variants, being c.337C>T (p.Arg113Ter) and c.394C>T (p.Arg132Ter) found in two families each. Regarding the carriers’ phenotypes, 12 of the 13 carriers had been diagnosed with polyposis and/or CRC. The age at the polyposis diagnosis and the number of polyps detected are highly diverse, ranging from 18 to 65 years of age and from 2 to more than 100 polyps. Three carriers had developed colon or rectal cancers, diagnosed at ages 23, 49 and 55 years, suggesting a relevant role in EOCRC. According to the limited data available, the diagnosis of a CRC does not seem to be directly associated with the number of polyps developed. Extracolonic cancers or other clinical manifestations are highly uncommon. The colonic lesions developed by carriers of *RNF43* pathogenic variants harbor somatic loss of heterozygosity or mutation in *RNF43* (2nd hits were identified in 100% (23/23) of the tumors analyzed); 50% (9/18) had CpG island methylator phenotype (CIMP). RNF43 functions as a tumor suppressor by exerting a negative feedback in the Wnt/β-catenin signaling pathway. Pathogenic variants in *RNF43* cause Wnt addiction, which makes *RNF43*-mutated tumors good responders to PORCN inhibitors [91], opening a window of opportunity for the treatment of tumors developed by *RNF43* carriers.

The frequency of pathogenic variants in *RNF43* among SPS patients, taking into account the two largest unselected serrated polyposis cohorts, is 1.76% (3/170) [89,90]; up to 2.63% (5/190) if all European cohorts are considered [86,89,90]. Due to the novelty and rarity of *RNF43*, reported studies using multigene panel testing in EOCRC did not include *RNF43*. Nevertheless, access to CanVar (https://canvar.icr.ac.uk/ (accessed on January 2021)), a public database that gathers whole-exome and -genome sequencing information of 1006 CRC patients diagnosed before age 60 years, showed that six patients carried rare germline missense variants, none of which was predicted pathogenic (REVEL > 0.5). No loss of function variants or variants affecting canonical splice sites were detected.

### 2.9. GREM1-Associated Mixed Polyposis

The term hereditary mixed polyposis syndrome originally referred to the presence of multiple polyps of different histological types (serrated lesions, conventional adenomas, and hamartomas) and/or the presence of individual polyps with various overlapping histological features (atypical juvenile and admixed histologic features) [92,93,94]. While the genetic cause remains elusive in most cases, germline variants in and upstream of *GREM1* have been identified in some affected individuals. In 2012, Jaeger et al. identified a 40-Kb duplication upstream of *GREM1* at chr15:30,752,231–30,792,051 (hg19), as the underlying cause of hereditary mixed polyposis in families of Ashkenazi Jewish origin [95]. The 40-kb duplication has been identified in 1:184 Ashkenazi Jewish individuals with personal or familial history of polyposis or CRC [96]. In addition to the founder Ashkenazi duplication, additional (non-Ashkenazi) variants identified include: a 16-Kb duplication affecting the *GREM1* 5′ regulatory region identified in a large family affected with polyposis of multiple polyp types, CRC, breast and gastric cancer [97]; a 24-Kb tandem duplication in the 5′ regulatory region identified in an Amsterdam-positive nonpolyposis CRC family, and a large duplication encompassing the whole *GREM1* gene in a patient with a CRC diagnosed at age 35 [98].

The data gathered to date indicate that *GREM1* duplications may occur in individuals with or without polyposis. The onset of the polyposis usually occurs in the adulthood (late 20s or older), although there are reports of polyps identified earlier in life. The phenotypes may overlap with familial adenomatous polyposis or Lynch syndrome (no polyps), and extracolonic tumors have been reported [96]. The nature of the alterations in this gene and the challenge of their detection by analyzing NGS data, as is the case of multigene panels, made the identification of *GREM1* duplications in the studied EOCRC patients difficult, preventing their reporting. Laduca et al. identified one *GREM1* pathogenic variant (nature not detailed in the original article) among 2366 EOCRC patients analyzed [11]. 

### 2.10. Clinical Consequences of having a Hereditary CRC Syndrome

Carrying a pathogenic variant in a cancer-predisposing syndrome has relevant consequences for the clinical management of the carrier. Consensus cancer surveillance guidelines and surgical recommendations, either prophylactic and/or therapeutic, for major colorectal cancer syndromes have been established and are periodically revised. For the rare syndromes that have been described more recently, such as those caused by pathogenic variants in *NTHL1*, *POLE* and *POLD1*, *RNF43* or *GREM1*, assessment of the clinical information from reported carriers is helping refine the corresponding clinical guidelines [57,98,99,100,101,102]. Together with periodic colonoscopy screening, chemoprevention might complete the precision prevention approach recommended for hereditary colorectal cancer and/or polyposis syndromes [103,104]. In particular, aspirin is recommended for Lynch syndrome carriers by most clinical guidelines [57], showing the most recent, long-term data from the CAPP2 study a robust reduction in incident colorectal cancers in Lynch syndrome carriers taking aspirin 600 mg/day. These data also show that aspirin uptake for a finite duration (mean 25 months) had sustained reduction in colorectal cancer incidence persisting for 20 years [105]. Nevertheless, additional data are expected that will define optimal dose (CAPP3 study), duration, and patient selection for Lynch syndrome-associated aspirin chemoprevention [106].

On the other hand, the choice of tumor chemotherapy treatment is influenced by specific tumor features that are characteristic of some hereditary syndromes [107]. Immune checkpoint blockade has proven effective in hypermutated tumors, including MMR- and *POLE/POLD1-* proofreading-deficient tumors [108,109], and recent preliminary data from a single case indicate that this treatment might also be effective for *MUTYH*-deficient tumors [110]. Other potential treatments for tumors developed in the context of a hereditary CRC syndrome include PORCN inhibitors for *RNF43*-deficient tumors [91], and the potential use of agents that induce BER-dependent DNA damage (e.g., Oxaliplatin or Temozolomide) might be considered for *NTHL1*- or *MUTYH*-deficient tumors (to be considered in future clinical assays).

In summary, recognition of a hereditary CRC syndrome in an eoCRC patient is extremely relevant for his/her clinical management, entitling specific surgical and treatment options depending on the mutated gene, and conditioning future clinical surveillance of the carrier and his/her relatives. Recent best practice pieces of advice for eoCRC patients recommend clinicians to counsel patients on the benefit of germline genetic testing in the presurgical period to inform which surgical options may be available to the patient; to use germline and somatic testing results to inform chemotherapeutic strategies; to offer, to the patient and other carrier family members, specific screening for CRC and extra-colonic cancers depending on the risks associated with the mutated gene [107].

## 3. EOCRC in Non-CRC Cancer Syndromes 

Data obtained from comprehensive multigene pan-cancer panels applied to familial and early-onset cancer, consistently demonstrate that many individuals who harbor pathogenic or likely pathogenic germline cancer susceptibility gene variants have clinical histories that fail to fulfill traditional, syndrome-specific guidelines, have atypical clinical phenotypes that a priori would not fit into the syndrome’s classical phenotype, or have germline pathogenic variants in more than one cancer-predisposing gene [111]. In this regard, EOCRC patients may also harbor pathogenic variants in genes not traditionally associated with CRC predisposition, such being the case for *BRCA1*, *BRCA2*, *TP53*, *ATM*, *CHEK2*, *PALB2*, *CDKN2A*, etc. (Table 1, [112,113]); these occurring in ≈2.5% of EOCRC cases.

## 4. Familial Aggregation of CRC in EOCRC 

While ≈13% (range: 9–26%) of EOCRC carry germline pathogenic variants in known cancer predisposition genes, ≈28% (range: 13–33%) have family history of CRC [5,6,8,9,10,11,114,115,116,117]. On the one hand, the absence of family history of CRC among EOCRC patients with a hereditary cancer syndrome ranges from 16.5% [6] to up to 60% [114], therefore a significant proportion of hereditary cases would not be suspected based on family history of this disease. On the other hand, a variable but relevant proportion of EOCRC patients have family history of CRC but no pathogenic variants in known hereditary cancer genes.

Large variability in the prevalence of family history of CRC is observed among studies focused on EOCRC: Stoffel et al. reported that 26% (111/430) of the EOCRC included in their study had at least one first-degree relative affected with CRC [6]], while this figure dropped to 6.7% in the EOCRC series analyzed by Chen et al. Considering family history of CRC as the presence of one 1st-, 2nd-, or 3rd-degree relative affected with the disease, O’Connell et al. [117] and Chen et al. [116] reported that 23–25% of EOCRC patients had familial CRC history and Stoffel et al. [6] reported that 57.2% if any relative with CRC is taken into account. The latter observed that the earlier the age at cancer diagnosis, the higher the chances of having family aggregation of the disease. Mork et al. reported that 18.6% of patients diagnosed before 35 years had at least one 1st or 2nd degree relative affected with CRC [8]. 

Individuals with family history of CRC (excluding hereditary syndromes) are known to be at increased risk for CRC. This risk is considered to be relevant in individuals with a first degree-relative (FDR) diagnosed below the age of 50 and in cases with two or more FDR with CRC [118]. In these situations, current guidelines recommend colonoscopy starting at age 40 years and performed every 5 years [118]. Family history of adenomas has been also associated with an increased CRC risk, however, the evidence is scarce and there is no clear consensus: whereas the ESGE guidelines [118] do not recommend screening in individuals with a FDR with adenomas, the U.S. Multi-Society Task Force of Colorectal Cancer [119], recommend screening for individuals with a FDR affected with an advanced adenoma before age 60 (colonoscopy starting at age 40 or 10 years before the age of diagnosis of the advanced adenoma in the FDR).

Unfortunately, screening guidelines for familial CRC are usually not followed. A major obstacle is the incomplete patient family history in medical records. Gupta et al. [120] in a cohort of 614 patients with CRC diagnosed between the ages of 40 and 49 years, reported that 98.4% (604 patients) should have been recommended to start screening at a younger age that their age at CRC diagnosis based on family history. Likewise, Stanich et al. observed that 97 (13.6%) of 713 EOCRC had family history of CRC, estimating that 82.5% (80/97) could have been diagnosed early and that 67% (65/97) would have had a potentially preventable CRC if screening guidelines based on family history would have been followed [114].

In summary, it is essential to optimize screening for CRC with early initiation of colonoscopy in cases with familial CRC history. Encouraging pedigree evaluation and stricter adherence to early CRC screening in this population is key to reduce EOCRC incidence and mortality. 

## 5. Low Risk Alleles and EOCRC

The fact that only ≈13% of EOCRC is explained by germline pathogenic variants in high penetrance genes suggests that another relevant fraction might be caused by the accumulation of genetic variants with low–moderate effect on cancer susceptibility.

Hundreds of common genetic variants associated with CRC susceptibility have been identified in the last years by means of genome-wide association studies (GWAS) (https://www.ebi.ac.uk/gwas (accessed on January 2021)). The use of a polygenic risk score (PRS) generated from 95 association signals could impact clinical decisions for CRC preventive screening in European populations [121]. Archambault et al. recently demonstrated that the 95 variant-based PRS correlated more strongly with EOCRC than with late-onset CRC, by testing 12,197 early-onset CRC patients (age < 50 years) and 95,865 CRC patients diagnosed at age ≥ 50 [13]. Patients in the highest PRS quartile had 3.7-fold increased risk for EOCRC (95% CI, 3.28–4.24) and a 2.9-fold increased risk for late-onset CRC (95% CI, 2.80–3.04), compared with the lowest quartile group. The association was stronger in patients without a first-degree family history of CRC, being the risk increased in this group 4.3-fold (95% CI, 3.61–5.01) vs. 2.9-fold (95% CI, 2.70–3.00) for early- and late-onset CRC, respectively, when the highest quartile was compared with the lowest.

It is expected that predictive models that implement a PRS in combination with environmental and lifestyle risk factors will, in the (near) future, be able to identify individuals at high risk of CRC that would benefit from preventive measures or other intervention strategies at earlier ages than the ones established in population-based screening programs or based on family history of CRC. 

## 6. Genetic Counseling and Genetic Testing Recommendations for EOCRC

Patients with EOCRC should be referred to a specialist service that includes genetic counseling and access to somatic and germline testing. Current guidelines recommend tumor screening for MMR deficiency for all CRC regardless of age and family history for Lynch syndrome diagnosis [99,122]. Germline testing should be offered to patients with MMR deficiency (with no evidence of *MLH1* promoter methylation) and those with polyposis. Since double somatic mutations in the MMR genes are presented in a meaningful proportion of patients with unexplained MMR deficiency, current guidelines already recommend paired germline and somatic MMR testing [122]. In this sense tumor MMR sequencing may be helpful for EOCRC showing MMR deficiency and no germline pathogenic variant detected [24]. The identification of double somatic mutations may be very helpful since these patients and their close relatives should be managed based on their family history as they are not Lynch syndrome. 

The data presented in this review (Table 1) suggest that a comprehensive multigene pan-cancer panel should be analyzed in every EOCRC patients, regardless of tumor MMR deficiency status. This recommendation is based on the prevalence of known hereditary CRC syndromes (≈10%), and also on the fact that ≈2.5% of EOCRC patients carry a germline pathogenic variant in a gene not traditionally linked to hereditary CRC. EOCRC patients with hereditary cancer syndromes should be managed according to established gene-specific surveillance guidelines. If possible, testing should occur prior to surgery since its results can affect surgical decision-making and therapeutic selection.

## 7. Conclusions

Epidemiological data indicate that EOCRC incidence is increasing at a global level, showing variability among countries. Established lifestyle cancer drivers such as diet, sedentary style, smoking, and obesity have been linked to EOCRC. Still unknown, probably unsuspected, nongenetic factors remain to be discovered. Hereditary syndromes are behind the pathogenicity of ≈13% of patients with EOCRC. In addition, a meaningful proportion of cases display either family history of CRC with no known high-penetrance genetic cause, or a germline pathogenic variant in genes not traditionally associated with CRC predisposition. 

Genetic testing using a multigene pan-cancer panel is recommended in all EOCRC patients, ideally in paired germline and tumor samples. On the other hand, preoperative and pretherapeutic testing is highly advisable, as it could give the best ability for informed surgical decision-making and for treatment selection, specifically when a pathogenic variant in an MMR gene, the exonuclease domain of *POLE* or *POLD1*, or a gene involved in homologous recombination (*BRCA1/2*, *ATM*, *PALB2*), is detected. In addition, the identification of a known hereditary cancer syndrome has relevant implications for cancer prevention in patients and relatives. 

Considering the prevalence of family history of CRC in EOCRC patients, improving adherence to current screening guidelines for individuals with CRC family history, would clearly have a significant impact on decreasing EOCRC. The implementation in predictive risk assessment of polygenic risk scores, together with family history information and lifestyle risk factors, will help better identify individuals at high risk of CRC that may benefit from cancer screening strategies or other interventions at earlier ages, also helping decrease the incidence of EOCRC.

## Figures and Tables

**Table 1:** Prevalence of germline pathogenic variants in hereditary cancer genes among early-onset colorectal cancer (EOCRC) patients. Studies including >100 EOCRC patients and analyzing multiple (>10) hereditary cancer genes or whole exomes are listed in the table.

Study [Population]	EOCRC Age Cutoff	No. Patients Analyzed	Testing Approach	Hereditary Cancer	Gene/Mutation Spectrum in CRC Genes ^a^ % P or LP Variant Carriers (n)	Gene/Mutation Spectrum in Other Cancer Genes ^a,b^ % P or LP Variant Carriers (n)
Laduca 2020 [11] [Different populations]	<50	Mean: 2672 (range: 986–4017)	Nine different multigene panels (5–49 genes evaluated)	362/4017 (9.0%)	5.3% MMR genes (213/3994) 0.5% *APC* (20/3884) 0.4% biallelic *MUTYH* (15/3953) 0.1% *SMAD4* (5/3881) 0.1% *BMPR1A* (3/3881) 0.0% *STK11* (1/3954) 0,0% *PTEN* (1/4014) 0.0% *GREM1* (1/2366)	1.5% *BRCA1/2* (21/1387) 1.1% *CHEK2* (43/3954) 1.0% *ATM* (14/,345) 0.2% *TP53* (9/4017) 0.1% *PALB2* (2/1350) 0.1% *BARD1* (1/1339) 0.3% *BRIP1* (4/1342) 0.1% *RAD51C* (1/1342) 0.2% *CDKN2A* (2/1228) 0.1% *SMARCA4* (1/986) 0.1% *NBN* (2/1339) 0.1% *CDH1* (2/3965) 0.1% *NF1* (1/1298)
Chubb 2016 [9] [UK]	≤55	1006	Exome sequencing	158/1006 (15.7%)	11% MMR genes (111) 1.9% *APC* (19) 0.9% biallelic *MUTYH* (9) 0.4% *POLE*/*POLD1* (4) 0.1% biallelic *NTHL1* (1)	0.9% *BRCA1*/2 (9) 0.3% *ATM* (3) 0.1% *TP53* (1) 0.1% *PALB2* (1)
Pearlman 2017 [5] [USA]	<50	450	25-gene panel	65/450 (14.4%)	8.2% MMR genes (37) 1.1% *APC* (5) 0.9% *APC* p.I1307K (4) 0.9% biallelic *MUTYH* (4) 0.2% *SMAD4* (1) 0.2% *APC*/*PMS2* (1)	0.45% *BRCA1* (2) 0.9% *BRCA2* (4) 0.7% *ATM* (3) 0.2% *ATM*/*CHEK2* (1) 0.45% *PALB2* (2) 0.2% *CDKN2A* (1)
DeRycke 2017 [10] [USA, Canada, Australia]	<50	333	36-gene panel	88/333 (26.4%)	13.5% MMR genes (45) 3.3% *APC* (11) 1.5% biallelic *MUTYH* (5) 0.9% *SMAD4* (3) 0.6% *BMPR1A* (2) 0.3% *PTEN* (1)	1.8% *CHEK2* (6) 0.6% *TP53* (2) 0.9% *CDH1* (3) 2.1% *RECQL5* (7) 0.6% *FLCN* (2) 0.3% biallelic *BLM* (1)
Stoffel 2018 [6] [USA]	<50	315	124-gene panel 67-gene panel	76/315 (24.1%)	17.8% MMR genes (56) 2.5% *APC* (8) 2.2% biallelic *MUTYH* (7) 0.6% *SMAD4* (2)	0.3% *BRCA1* (1) 0.3% *CHEK2* (1) 0.3% *TP53* (1)
Jiang 2020 [12] [China]	<50	261	14 genes analyzed by target sequencing	47/261 (18%)	15.7% MMR genes (41) 1.5% *APC* (4) 0.4% biallelic *MUTYH* (1)^c^ 0.4% *STK11* (1)	
Mork 2015 [8] [USA]	<35	205	Variety of germline tests	41/193 (21.2%)	11.9 % MMR genes (23) 6.7% *APC* (13) 1% biallelic *MUTYH* (2) 1% biallelic *MSH6*/*PMS2* (2)	0.5% *TP53* (1)
**Total**		**6587**		**837/6587 (12.7%)**	**682/6587 (10.4%)**	**155/6587 (2.4%)**

^a^ Pathogenic and likely pathogenic variants, as reported by the authors, are considered in the calculations. ^b^ Carriers of disruptive variants (frameshift, stop-gain and start-loss variants) are considered for non-CRC cancer genes in the study by Chubb et al. ^c^ Considered biallelic, although there are no specific details in the original article.

## Data Availability

No new data were created or analyzed in this study.

## References

[1] Stoffel E.M., Murphy C.C. (2020). Epidemiology and Mechanisms of the Increasing Incidence of Colon and Rectal Cancers in Young Adults. Gastroenterology.

[2] Siegel R.L., Torre L.A., Soerjomataram I., Hayes R.B., Bray F., Weber T.K., Jemal A. (2019). Global patterns and trends in colorectal cancer incidence in young adults. Gut.

[3] Jasperson K.W., Tuohy T.M., Neklason D.W., Burt R.W. (2010). Hereditary and familial colon cancer. Gastroenterology.

[4] Patel S.G., Boland C.R. (2020). Colorectal Cancer in Persons Under Age 50: Seeking Causes and Solutions. Gastrointest Endosc. Clin. N. Am..

[5] Pearlman R., Frankel W.L., Swanson B., Zhao W., Yilmaz A., Miller K., Bacher J., Bigley C., Nelsen L., Goodfellow P.J. (2017). Prevalence and Spectrum of Germline Cancer Susceptibility Gene Mutations Among Patients With Early-Onset Colorectal Cancer. JAMA Oncol..

[6] Stoffel E.M., Koeppe E., Everett J., Ulintz P., Kiel M., Osborne J., Williams L., Hanson K., Gruber S.B., Rozek L.S. (2018). Germline Genetic Features of Young Individuals With Colorectal Cancer. Gastroenterology.

[7] Chang D.T., Pai R.K., A Rybicki L., A Dimaio M., Limaye M., Jayachandran P., Koong A.C., A Kunz P., A Fisher G., Ford J.M. (2012). Clinicopathologic and molecular features of sporadic early-onset colorectal adenocarcinoma: An adenocarcinoma with frequent signet ring cell differentiation, rectal and sigmoid involvement, and adverse morphologic features. Mod. Pathol..

[8] Mork M.E., You Y.N., Ying J., Bannon S.A., Lynch P.M., Rodriguez-Bigas M.A., Vilar E. (2015). High Prevalence of Hereditary Cancer Syndromes in Adolescents and Young Adults With Colorectal Cancer. J. Clin. Oncol..

[9] Chubb D., Broderick P., Dobbins S.E., Frampton M., Kinnersley B., Penegar S., Price A., Ma Y.P., Sherborne A.L., Palles C. (2016). Rare disruptive mutations and their contribution to the heritable risk of colorectal cancer. Nat. Commun..

[10] DeRycke M.S., Gunawardena S., Balcom J.R., Pickart A.M., Waltman L.A., French A.J., McDonnell S., Riska S.M., Fogarty Z.C., Larson M.C. (2017). Targeted sequencing of 36 known or putative colorectal cancer susceptibility genes. Mol. Genet. Genom. Med..

[11] LaDuca H., Polley E.C., Yussuf A., Hoang L., Bs S.G., Hart S.N., Yadav S., Hu C., Na Ms J., Goldgar D.E. (2020). A clinical guide to hereditary cancer panel testing: Evaluation of gene-specific cancer associations and sensitivity of genetic testing criteria in a cohort of 165,000 high-risk patients. Genet. Med..

[12] Jiang T., Wang F., Wang Y., Hu J., Ding P., Lin J., Pan Z., Chen G., Shao J., Xu R. (2020). Germline mutational profile of Chinese patients under 70 years old with colorectal cancer. Cancer Commun..

[13] Archambault A.N., Su Y.R., Jeon J., Thomas M., Lin Y., Conti D.V., Win A.K., Sakoda L.C., Lansdorp-Vogelaar I., Peterse E.F.P. (2020). Cumulative Burden of Colorectal Cancer-Associated Genetic Variants Is More Strongly Associated with Early-Onset vs Late-Onset Cancer. Gastroenterology.

[14] Dominguez-Valentin M., Sampson J.R., Seppälä T.T., Ten Broeke S.W., Plazzer J.P., Nakken S., Engel C., Aretz S., Jenkins M.A., Sunde L. (2020). Cancer risks by gene, age, and gender in 6350 carriers of pathogenic mismatch repair variants: Findings from the Prospective Lynch Syndrome Database. Genet. Med..

[15] Vasen H.F., Boland C.R. (2005). Progress in genetic testing, classification, and identification of Lynch syndrome. JAMA.

[16] Moreira L., Balaguer F., Lindor N., De La Chapelle A., Hampel H., Aaltonen L.A., Hopper J.L., Le Marchand L., Gallinger S., Newcomb P.A. (2012). Identification of Lynch Syndrome Among Patients With Colorectal Cancer. JAMA.

[17] Lynch H.T., Smyrk T.C., Watson P., Lanspa S.J., Lynch J.F., Lynch P.M., Cavalieri R., Boland C. (1993). Genetics, natural history, tumor spectrum, and pathology of hereditary nonpolyposis colorectal cancer: An updated review. Gastroenterology.

[18] Mendelsohn R.B., Herzog K., Shia J., Rahaman N., Stadler Z.K., Shike M. (2017). Molecular Screening for Lynch Syndrome in Young Patients With Colorectal Adenomas. Clin. Colorectal. Cancer.

[19] Lynch H.T., Lynch J.F., Lynch P.M., Attard T. (2008). Hereditary colorectal cancer syndromes: Molecular genetics, genetic counseling, diagnosis and management. Fam. Cancer.

[20] Giardiello F.M., Allen J.I., Axilbund J.E., Boland C.R., Burke C.A., Burt R.W., Church J.M., Dominitz J.A., Johnson D.A., Kaltenbach T. (2014). Guidelines on Genetic Evaluation and Management of Lynch Syndrome: A Consensus Statement by the US Multi-Society Task Force on Colorectal Cancer. Gastroenterology.

[21] Hendriks Y.M., Wagner A., Morreau H., Menko F., Stormorken A., Quehenberger F., Sandkuijl L., Møller P., Genuardi M., van Houwelingen H. (2004). Cancer risk in hereditary nonpolyposis colorectal cancer due to MSH6 mutations: Impact on counseling and surveillance. Gastroenterology.

[22] Senter L., Clendenning M., Sotamaa K., Hampel H., Green J., Potter J.D., Lindblom A., Lagerstedt K., Thibodeau S.N., Lindor N.M. (2008). The Clinical Phenotype of Lynch Syndrome Due to Germ-Line PMS2 Mutations. Gastroenterology.

[23] Jenkins M.A., Baglietto L., Dowty J.G., Van Vliet C.M., Smith L., Mead L.J., Macrae F.A., John D.J.B.S., Jass J.R., Giles G.G. (2006). Cancer Risks For Mismatch Repair Gene Mutation Carriers: A Population-Based Early Onset Case-Family Study. Clin. Gastroenterol. Hepatol..

[24] Pearlman R., Haraldsdottir S., de la Chapelle A., Jonasson J.G., Liyanarachchi S., Frankel W.L., Rafnar T., Stefansson K., Pritchard C.C., Hampel H. (2019). Clinical characteristics of patients with colorectal cancer with double somatic mismatch repair mutations compared with Lynch syndrome. J. Med. Genet..

[25] Hampel H., Frankel W.L., Martin E., Arnold M., Khanduja K., Kuebler P., Nakagawa H., Sotamaa K., Prior T.W., Westman J. (2005). Screening for the Lynch syndrome (hereditary nonpolyposis colorectal cancer). N. Engl. J. Med..

[26] Seguí N., Navarro M., Pineda M., Köger N., Bellido F., González S., Campos O., Iglesias S., Valdés-Mas R., López-Doriga A. (2015). Exome sequencing identifies MUTYH mutations in a family with colorectal cancer and an atypical phenotype. Gut.

[27] Morak M., Heidenreich B., Keller G., Hampel H., Laner A., De La Chapelle A., Holinski-Feder E. (2014). Biallelic MUTYH mutations can mimic Lynch syndrome. Eur. J. Hum. Genet..

[28] A Elsayed F., Kets C.M., Ruano D., Akker B.V.D., Mensenkamp A.R., Schrumpf M., Nielsen M., Wijnen J.T., Tops C.M., Ligtenberg M.J. (2015). Germline variants in POLE are associated with early onset mismatch repair deficient colorectal cancer. Eur. J. Hum. Genet..

[29] Rayner E., van Gool I.C., Palles C., Kearsey S.E., Bosse T., Tomlinson I., Church D.N. (2016). A panoply of errors: Polymerase proofreading domain mutations in cancer. Nat. Rev. Cancer.

[30] Alexandrov L.B., Kim J., Haradhvala N.J., Huang M.N., Tian Ng A.W., Wu Y., Boot A., Covington K.R., Gordenin D.A., Bergstrom E.N. (2020). The repertoire of mutational signatures in human cancer. Nature.

[31] Limburg P.J., Harmsen W.S., Chen H.H., Gallinger S., Haile R.W., Baron J.A., Casey G., Woods M.O., Thibodeau S.N., Lindor N.M. (2011). Prevalence of Alterations in DNA Mismatch Repair Genes in Patients With Young-Onset Colorectal Cancer. Clin. Gastroenterol. Hepatol..

[32] Giráldez M.D., Balaguer F., Bujanda L., Cuatrecasas M., Muñoz J., Alonso-Espinaco V., Larzabal M., Petit A., Gonzalo V., Ocaña T. (2010). MSH6 and MUTYH deficiency is a frequent event in early-onset colorectal cancer. Clin. Cancer Res..

[33] Wimmer K., Kratz C.P., Vasen H.F., Caron O., Colas C., Entz-Werle N., Gerdes A.M., Goldberg Y., Ilencikova D., Muleris M. (2014). Diagnostic criteria for constitutional mismatch repair deficiency syndrome: Suggestions of the European consortium ‘care for CMMRD’ (C4CMMRD). J. Med. Genet..

[34] Suerink M., Wimmer K., Brugieres L., Colas C., Gallon R., Ripperger T., Benusiglio E.M., Bleiker A., Ghorbanoghli Z., Goldberg Y. (2020). Report of the fifth meeting of the European Consortium ‘Care for CMMRD’ (C4CMMRD), Leiden, The Netherlands, July 6th 2019. Fam. Cancer.

[35] Terradas M., Capellá G., Valle L. (2020). Dominantly Inherited Hereditary Nonpolyposis Colorectal Cancer Not Caused by MMR Genes. J. Clin. Med..

[36] Nieminen T.T., O’Donohue M.F., Wu Y., Lohi H., Scherer S.W., Paterson A.D., Ellonen P., Abdel-Rahman W.M., Valo S., Mecklin J.P. (2014). Germline mutation of RPS20, encoding a ribosomal protein, causes predisposition to hereditary nonpolyposis colorectal carcinoma without DNA mismatch repair deficiency. Gastroenterology.

[37] Broderick P., Dobbins S.E., Chubb D., Kinnersley B., Dunlop M.G., Tomlinson I., Houlston R.S. (2017). Validation of Recently Proposed Colorectal Cancer Susceptibility Gene Variants in an Analysis of Families and Patients—a Systematic Review. Gastroenterology.

[38] Belhadj S., Terradas M., Munoz-Torres P.M., Aiza G., Navarro M., Capellá G., Valle L. (2020). Candidate genes for hereditary colorectal cancer: Mutational screening and systematic review. Hum. Mutat..

[39] Thompson B.A., Snow A.K., Koptiuch C., Kohlmann W.K., Mooney R., Johnson S., Huff C.D., Yu Y., Teerlink C.C., Feng B. (2020). A novel ribosomal protein S20 variant in a family with unexplained colorectal cancer and polyposis. Clin. Genet..

[40] Ma H., Brosens L.A.A., Offerhaus G.J.A., Giardiello F.M., de Leng W.W.J., Montgomery E.A. (2018). Pathology and genetics of hereditary colorectal cancer. Pathology.

[41] Dinarvand P., Davaro E.P., Doan J.V., Ising M.E., Evans N.R., Phillips N.J., Lai V., Guzman M.A. (2019). Familial Adenomatous Polyposis Syndrome: An Update and Review of Extraintestinal Manifestations. Arch. Pathol. Lab. Med..

[42] Morin P.J. (2019). Colorectal cancer: The APC-lncRNA link. J. Clin. Invest.

[43] Zhang J., Li Z., Huang X., Ye J. (2016). [Clinical and molecular characteristics of a child with familial adenomatous polyposis]. Zhonghua Er Ke Za Zhi.

[44] Palles C., Cazier J.B., Howarth K.M., Domingo E., Jones A.M., Broderick P., Kemp Z., Spain S.L., Guarino E., Guarino Almeida E. (2013). Germline mutations affecting the proofreading domains of POLE and POLD1 predispose to colorectal adenomas and carcinomas. Nat. Genet..

[45] Palles C., Latchford A., Valle L., Valle L., Gruber S.B., Capellá G. (2018). Adenomatous polyposis syndromes: Polymerase proofreading-associated polyposis. Hereditary Colorectal Cancer Genetic Basis and Clinical Implications.

[46] Wimmer K., Beilken A., Nustede R., Ripperger T., Lamottke B., Ure B., Steinmann D., Reineke-Plaass T., Lehmann U., Zschocke J. (2017). A novel germline POLE mutation causes an early onset cancer prone syndrome mimicking constitutional mismatch repair deficiency. Fam. Cancer.

[47] Lindsay H., Scollon S., Reuther J., Voicu H., Rednam S.P., Lin F.Y., Fisher K.E., Chintagumpala M., Adesina A.M., Parsons D.W. (2019). Germline POLE mutation in a child with hypermutated medulloblastoma and features of constitutional mismatch repair deficiency. Cold Spring Harb. Mol. Case Stud..

[48] van Gool I.C., Bosse T., Church D.N. (2016). proofreading mutation, immune response and prognosis in endometrial cancer. Oncoimmunology.

[49] Mur P., Ms S.G.-M., Del Valle J., Ms L.M.-P., Vidal A., Pineda M., Cinnirella G., Ms E.M.-R., Pons T., López-Doriga A. (2020). Role of POLE and POLD1 in familial cancer. Genet. Med..

[50] Achatz M.I., Porter C.C., Brugières L., Druker H., Frebourg T., Foulkes W.D., Kratz C.P., Kuiper R.P., Hansford J.R., Hernandez H.S. (2017). Cancer Screening Recommendations and Clinical Management of Inherited Gastrointestinal Cancer Syndromes in Childhood. Clin. Cancer Res..

[51] Nielsen M., Joerink-van de Beld M.C., Jones N., Vogt S., Tops C.M., Vasen H.F., Sampson J.R., Aretz S., Hes F.J. (2009). Analysis of MUTYH genotypes and colorectal phenotypes in patients With MUTYH-associated polyposis. Gastroenterology.

[52] Castillejo A., Vargas G., Castillejo M.I., Navarro M., Barberá V.M., González S., Hernández-Illán E., Brunet J., Cajal T.R.Y., Balmaña J. (2014). Prevalence of germline MUTYH mutations among Lynch-like syndrome patients. Eur. J. Cancer.

[53] Pilati C., Shinde J., Alexandrov L.B., Assie G., Andre T., Helias-Rodzewicz Z., Ducoudray R., Le Corre D., Zucman-Rossi J., Emile J.F. (2017). Mutational signature analysis identifies MUTYH deficiency in colorectal cancers and adrenocortical carcinomas. J. Pathol..

[54] Viel A., Bruselles A., Meccia E., Fornasarig M., Quaia M., Canzonieri V., Policicchio E., Urso E.D., Agostini M., Genuardi M. (2017). A Specific Mutational Signature Associated with DNA 8-Oxoguanine Persistence in MUTYH-defective Colorectal Cancer. EBioMedicine.

[55] Win A.K., Dowty J.G., Cleary S.P., Kim H., Buchanan D.D., Young J.P., Clendenning M., Rosty C., MacInnis R.J., Giles G.G. (2014). Risk of colorectal cancer for carriers of mutations in MUTYH, with and without a family history of cancer. Gastroenterology.

[56] Theodoratou E., Campbell H., Tenesa A., Houlston R., Webb E., Lubbe S., Broderick P., Gallinger S., Croitoru E.M., Jenkins M.A. (2010). A large-scale meta-analysis to refine colorectal cancer risk estimates associated with MUTYH variants. Br. J. Cancer.

[57] National Comprehensive Cancer Network NCCN Clinical Practice Guidelines in Oncology (version 1.2020). Genetic/Familial High-Risk Assessment: Colorectal [Internet]. https://www.nccn.org/professionals/physician_gls/pdf/genetics_colon.pdf.

[58] A Weren R.D., Ligtenberg M.J.L., Kets C.M., De Voer R.M., Verwiel E.T.P., Spruijt L., Zelst-Stams W.A.G.V., Jongmans M.C., Gilissen C., Hehir-Kwa J.Y. (2015). A germline homozygous mutation in the base-excision repair gene NTHL1 causes adenomatous polyposis and colorectal cancer. Nat. Genet..

[59] Kuiper R.P., Nielsen M., De Voer R.M., Hoogerbrugge N., Adam M.P., Ardinger H.H., Pagon R.A., Wallace S.E., Bean L.J.H., Stephens K., Amemiya A. (2020). NTHL1 Tumor Syndrome.

[60] Rivera B., Castellsagué E., Bah I., van Kempen L.C., Foulkes W.D. (2015). Biallelic NTHL1 Mutations in a Woman with Multiple Primary Tumors. N. Engl. J. Med..

[61] Belhadj S., Mur P., Navarro M., González S., Moreno V., Capellá G., Valle L. (2017). Delineating the Phenotypic Spectrum of the NTHL1-Associated Polyposis. Clin. Gastroenterol. Hepatol..

[62] Fostira F., Kontopodis E., Apostolou P., Fragkaki M., Androulakis N., Yannoukakos D., Konstantopoulou I., Saloustros E. (2018). Extending the clinical phenotype associated with biallelic NTHL1 germline mutations. Clin. Genet..

[63] Belhadj S., Quintana I., Mur P., Munoz-Torres P.M., Alonso M.H., Navarro M., Terradas M., Piñol V., Brunet J., Moreno V. (2019). NTHL1 biallelic mutations seldom cause colorectal cancer, serrated polyposis or a multi-tumor phenotype, in absence of colorectal adenomas. Sci. Rep..

[64] Altaraihi M., Gerdes A.M., Wadt K. (2019). A new family with a homozygous nonsense variant in NTHL1 further delineated the clinical phenotype of NTHL1-associated polyposis. Hum. Genome Var.

[65] Groves A., Gleeson M., Spigelman A.D. (2019). NTHL1-associate polyposis: First Australian case report. Fam. Cancer.

[66] Grolleman J.E., de Voer R.M., Elsayed F.A., Nielsen M., Weren R.D.A., Palles C., Ligtenberg M.J.L., Vos J.R., Ten Broeke S.W., de Miranda N.F.C.C. (2019). Mutational Signature Analysis Reveals NTHL1 Deficiency to Cause a Multi-tumor Phenotype. Cancer Cell.

[67] Weren R.D., Ligtenberg M.J., Geurts van Kessel A., De Voer R.M., Hoogerbrugge N., Kuiper R.P. (2018). NTHL1 and MUTYH polyposis syndromes: Two sides of the same coin?. J. Pathol..

[68] Terradas M., Munoz-Torres P.M., Belhadj S., Aiza G., Navarro M., Brunet J., Capellá G., Valle L. (2019). Contribution to colonic polyposis of recently proposed predisposing genes and assessment of the prevalence of NTHL1 - and MSH3 -associated polyposes. Hum. Mutat..

[69] Drost J., van Boxtel R., Blokzijl F., Mizutani T., Sasaki N., Sasselli V., de Ligt J., Behjati S., Grolleman J.E., van Wezel T. (2017). Use of CRISPR-modified human stem cell organoids to study the origin of mutational signatures in cancer. Science.

[70] Hearle N., Schumacher V., Menko F.H., Olschwang S., Boardman L.A., Gille J.J., Keller J.J., Westerman A.M., Scott R.J., Lim W. (2006). Frequency and spectrum of cancers in the Peutz-Jeghers syndrome. Clin. Cancer Res..

[71] Latchford A., Cohen S., Auth M., Scaillon M., Viala J., Daniels R., Talbotec C., Attard T., Durno C., Hyer W. (2019). Management of Peutz-Jeghers Syndrome in Children and Adolescents: A Position Paper From the ESPGHAN Polyposis Working Group. J. Pediatr. Gastroenterol. Nutr..

[72] Volikos E., Robinson J., Aittomäki K., Mecklin J.P., Järvinen H., Westerman A.M., de Rooji F.W., Vogel T., Moeslein G., Launonen V. (2006). LKB1 exonic and whole gene deletions are a common cause of Peutz-Jeghers syndrome. J. Med. Genet..

[73] Gammon A., Jasperson K., Kohlmann W., Burt R.W. (2009). Hamartomatous polyposis syndromes. Best Pr. Res. Clin. Gastroenterol..

[74] Kidambi T.D., Kohli D.R., Samadder N.J., Singh A. (2019). Hereditary Polyposis Syndromes. Curr. Treat Options Gastroenterol..

[75] Larsen Haidle J., Howe J.R., Adam M.P., Ardinger H.H., Pagon R.A., Wallace S.E., Bean L.J.H., Mirzaa G., Amemiya A. (1993). Juvenile Polyposis Syndrome.

[76] Tan M.H., Mester J.L., Ngeow J., Rybicki L.A., Orloff M.S., Eng C. (2012). Lifetime cancer risks in individuals with germline PTEN mutations. Clin. Cancer Res..

[77] Tischkowitz M., Colas C., Pouwels S., Hoogerbrugge N., Group P.G.D., Genturis E.R.N. (2020). Cancer Surveillance Guideline for individuals with PTEN hamartoma tumour syndrome. Eur. J. Hum. Genet..

[78] Pilarski R. (2009). Cowden syndrome: A critical review of the clinical literature. J. Genet. Couns..

[79] Carballal S., Rodriguez-Alcalde D., Moreira L., Hernandez L., Rodriguez L., Rodriguez-Moranta F., Gonzalo V., Bujanda L., Bessa X., Poves C. (2016). Colorectal cancer risk factors in patients with serrated polyposis syndrome: A large multicentre study. Gut.

[80] Ijspeert J.E.G., Rana S.A.Q., Atkinson N.S.S., Van Herwaarden Y.J., Bastiaansen B.A.J., E Van Leerdam M., Sanduleanu S., Bisseling T.M., Spaander M.C.W., Clark S.K. (2017). Clinical risk factors of colorectal cancer in patients with serrated polyposis syndrome: A multicentre cohort analysis. Gut.

[81] Rodríguez-Alcalde D., Carballal S., Moreira L., Hernández L., Rodríguez-Alonso L., Rodríguez-Moranta F., Gonzalo V., Bujanda L., Bessa X., Poves C. (2019). High incidence of advanced colorectal neoplasia during endoscopic surveillance in serrated polyposis syndrome. Endoscopy.

[82] Bleijenberg A.G., Ijspeert J.E., Van Herwaarden Y.J., Carballal S., Pellisé M., Jung G., Bisseling T.M., Nagetaal I.D., E Van Leerdam M., Van Lelyveld N. (2019). Personalised surveillance for serrated polyposis syndrome: Results from a prospective 5-year international cohort study. Gut.

[83] IJspeert J.E.G., Bevan R., Senore C., Kaminski M.F., Kuipers E.J., Mroz A., Bessa X., Cassoni P., Hassan C., Repici A. (2017). Detection rate of serrated polyps and serrated polyposis syndrome in colorectal cancer screening cohorts: A European overview. Gut.

[84] Rivero-Sanchez L., Lopez-Ceron M., Carballal S., Moreira L., Bessa X., Serradesanferm A., Pozo A., Augé J.M., Ocaña T., Sánchez A. (2017). Reassessment colonoscopy to diagnose serrated polyposis syndrome in a colorectal cancer screening population. Endoscopy.

[85] Dekker E., Bleijenberg A., Balaguer F., IJspeert J.E., Bleijenberg A.G., Pellisé M., Carballal S., Rivero L., Latchford A. (2020). Update on the World Health Organization Criteria for Diagnosis of Serrated Polyposis Syndrome. Gastroenterology.

[86] Gala M.K., Mizukami Y., Le L.P., Moriichi K., Austin T., Yamamoto M., Lauwers G.Y., Bardeesy N., Chung D.C. (2014). Germline Mutations in Oncogene-Induced Senescence Pathways Are Associated With Multiple Sessile Serrated Adenomas. Gastroenterology.

[87] Taupin D., Lam W., Rangiah D., McCallum L., Whittle B., Zhang Y., Andrews D., Field M., Goodnow C.C., Cook M.C. (2015). A deleterious RNF43 germline mutation in a severely affected serrated polyposis kindred. Hum. Genome Var..

[88] Yan H.H., Lai J.C., Ho S.L., Leung W.K., Law W.L., Lee J.F., Chan A.K.W., Tsui W.Y., Chan A.S.Y., Lee B.C.H. (2016). RNF43 germline and somatic mutation in serrated neoplasia pathway and its association with BRAF mutation. Gut.

[89] Buchanan D.D., Clendenning M., Zhuoer L., Stewart J.R., Joseland S., Woodall S., Arnold J., Semotiuk K., Aronson M., Holter S. (2017). Lack of evidence for germline RNF43 mutations in patients with serrated polyposis syndrome from a large multinational study. Gut.

[90] Quintana I., Mejías-Luque R., Terradas M., Navarro M., Piñol V., Mur P., Belhadj S., Grau E., Darder E., Solanes A. (2018). Evidence suggests that germline RNF43 mutations are a rare cause of serrated polyposis. Gut.

[91] Yu J., Mohamed Yuso P.A.B., Woutersen D.T.J., Goh P., Harmston N., Smits R., Harmston N., Smits R., Epstein D.M., Virshup D.M. (2020). The functional landscape of patient-derived RNF43 mutations predicts sensitivity to Wnt inhibiton. Cancer Res..

[92] Jaeger E., Woodford-Richens K., Lockett M., Rowan A., Sawyer E., Heinimann K., Rozen P., Murday V., Whitelaw S., Ginsberg A. (2003). An Ancestral Ashkenazi Haplotype at the HMPS/CRAC1 Locus on 15q13–q14 Is Associated with Hereditary Mixed Polyposis Syndrome. Am. J. Hum. Genet..

[93] Whitelaw S.C., A Murday V., Tomlinson I.P., Thomas H.J., Cottrell S., Ginsberg A., Bukofzer S., Hodgson S.V., Skudowitz R.B., Jass J.R. (1997). Clinical and molecular features of the hereditary mixed polyposis syndrome. Gastroenterology.

[94] Thomas H.J., Whitelaw S.C., Cottrell S.E., Murday V.A., Tomlinson I.P., Markie D., Jones T., Bishop D.T., Hodgson S.V., Sheer D. (1996). Genetic mapping of hereditary mixed polyposis syndrome to chromosome 6q. Am. J. Hum. Genet..

[95] Jaeger E., Leedham S.J., Lewis A., Segditsas S., Becker M., Cuadrado P.R., Davis H., Kaur K., Heinimann K., Howarth K. (2012). Hereditary mixed polyposis syndrome is caused by a 40-kb upstream duplication that leads to increased and ectopic expression of the BMP antagonist GREM1. Nat. Genet..

[96] Lieberman S., Walsh T., Schechter M., Adar T., Goldin E., Beeri R., Baris H., Avi L.B., Half E., Lerer I. (2017). Features of Patients With Hereditary Mixed Polyposis Syndrome Caused by Duplication of GREM1 and Implications for Screening and Surveillance. Gastroenterology.

[97] Rohlin A., Eiengard F., Lundstam U., Zagoras T., Nilsson S., Edsjo A., Pedersen J., Svensson J., Skullman S., Karlsson B.G. (2016). GREM1 and POLE variants in hereditary colorectal cancer syndromes. Genes Chromosomes Cancer.

[98] Venkatachalam R., Verwiel E.T., Kamping E.J., Hoenselaar E., Gorgens H., Schackert H.K., van Krieken J.H.J.M., Ligtenberg M.J.L., Hoogerbrugge N., van Kessel A.G. (2011). Identification of candidate predisposing copy number variants in familial and early-onset colorectal cancer patients. Int. J. Cancer.

[99] Monahan K.J., Bradshaw N., Dolwani S., Desouza B., Dunlop M.G., E East J., Ilyas M., Kaur A., Lalloo F., Latchford A. (2019). Guidelines for the management of hereditary colorectal cancer from the British Society of Gastroenterology (BSG)/Association of Coloproctology of Great Britain and Ireland (ACPGBI)/United Kingdom Cancer Genetics Group (UKCGG). Gut.

[100] Idos G., Valle L., Adam M.P., Ardinger H.H., Pagon R.A., Wallace S.E., Bean L.J.H., Mirzaa G., Amemiya A. (1993). Lynch Syndrome.

[101] Seppälä T.T., Latchford A., Negoi I., Soares A.S., Jimenez-Rodriguez R., Sánchez-Guillén L., Evans D.G., Ryan N., Crosbie E.J., Dominguez-Valentin M. (2020). European guidelines from the EHTG and ESCP for Lynch syndrome: An updated third edition of the Mallorca guidelines based on gene and gender. Br. J. Surg..

[102] Herzig D., Hardimann K., Weiser M., You N., Paquette I., Feingold D.L., Steele S.R. (2017). The American Society of Colon and Rectal Surgeons Clinical Practice Guidelines for the Management of Inherited Polyposis Syndromes. Dis. Colon Rectum.

[103] Valle L., Vilar E., Tavtigian S.V., Stoffel E.M. (2019). Genetic predisposition to colorectal cancer: Syndromes, genes, classification of genetic variants and implications for precision medicine. J. Pathol..

[104] Ulusan A.M., Rajendran P., Dashwood W.M., Yavuz O.F., Kapoor S., Gustafson T.A., Savage M.I., Brown P.H., Sei S., Mohammed A. (2021). Optimization of Erlotinib Plus Sulindac Dosing Regimens for Intestinal Cancer Prevention in an Apc-Mutant Model of Familial Adenomatous Polyposis (FAP). Cancer Prev. Res..

[105] Burn J., Sheth H., Elliott F., Reed L., Macrae F., Mecklin J.-P., Möslein G., E McRonald F., Bertario L., Evans D.G. (2020). Cancer prevention with aspirin in hereditary colorectal cancer (Lynch syndrome), 10-year follow-up and registry-based 20-year data in the CAPP2 study: A double-blind, randomised, placebo-controlled trial. Lancet.

[106] Yurgelun M.B., Chan A.T. (2020). Aspirin for Lynch syndrome: A legacy of prevention. Lancet.

[107] Boardman L.A., Vilar E., You Y.N., Samadder J. (2020). AGA Clinical Practice Update on Young Adult-Onset Colorectal Cancer Diagnosis and Management: Expert Review. Clin. Gastroenterol. Hepatol..

[108] André T., Shiu K.-K., Kim T.W., Jensen B.V., Jensen L.H., Punt C., Smith D., Garcia-Carbonero R., Benavides M., Gibbs P. (2020). Pembrolizumab in Microsatellite-Instability–High Advanced Colorectal Cancer. New Engl. J. Med..

[109] Lau D., Kalaitzaki E., Church D.N., Pandha H., Tomlinson I., Annels N., Gerlinger M., Sclafani F., Smith G., Begum R. (2020). Rationale and design of the POLEM trial: Avelumab plus fluoropyrimidine-based chemotherapy as adjuvant treatment for stage III mismatch repair deficient or POLE exonuclease domain mutant colon cancer: A phase III randomised study. ESMO Open.

[110] Volkov N.M., Yanus G.A., Ivantsov A.O., Moiseenko F.V., Matorina O.G., Bizin I.V., Moiseyenko V.M., Imyanitov E.N. (2019). Efficacy of immune checkpoint blockade in MUTYH-associated hereditary colorectal cancer. Investig. New Drugs.

[111] Boland P.M., Yurgelun M.B., Boland C.R. (2018). Recent progress in Lynch syndrome and other familial colorectal cancer syndromes. CA Cancer J. Clin..

[112] Valle L., de Voer R.M., Goldberg Y., Sjursen W., Försti A., Ruiz-Ponte C., Caldés T., Garré P., Olsen M.F., Nordling M. (2019). Update on genetic predisposition to colorectal cancer and polyposis. Mol. Aspects Med..

[113] Terradas M., Mur P., Belhadj S., Woodward E.R., Burghel G.J., Munoz-Torres P.M., Quintana I., Navarro M., Brunet J., Lazaro C. (2020). TP53, a gene for colorectal cancer predisposition in the absence of Li-Fraumeni-associated phenotypes. Gut.

[114] Stanich P.P., Pelstring K.R., Hampel H., Pearlman R. (2020). A High Percentage of Early-Age Onset Colorectal Cancer is Potentially Preventable. Gastroenterology.

[115] Chen F.W., Sundaram V., Chew T.A., Ladabaum U. (2017). Advanced-Stage Colorectal Cancer in Persons Younger Than 50 Years Not Associated With Longer Duration of Symptoms or Time to Diagnosis. Clin. Gastroenterol. Hepatol..

[116] Chen F.W., Sundaram V., Chew T.A., Ladabaum U. (2017). Low Prevalence of Criteria for Early Screening in Young-Onset Colorectal Cancer. Am. J. Prev. Med..

[117] O’Connell J.B., Maggard M.A., Livingston E.H., Yo C.K. (2004). Colorectal cancer in the young. Am. J. Surg..

[118] van Leerdam M.E., Roos V.H., van Hooft J.E., Balaguer F., Dekker E., Kaminski M.F., Latchford A., Neumann H., Ricciardiello L., Rupińska M. (2019). Endoscopic management of Lynch syndrome and of familial risk of colorectal cancer: European Society of Gastrointestinal Endoscopy (ESGE) Guideline. Endoscopy.

[119] Rex D.K., Boland C.R., Dominitz J.A., Giardiello F.M., Johnson D.A., Kaltenbach T., Levin T.R., Lieberman D., Robertson D.J. (2017). Colorectal Cancer Screening: Recommendations for Physicians and Patients From the U.S. Multi-Society Task Force on Colorectal Cancer. Gastroenterology.

[120] Gupta S., Bharti B., Ahnen D.J., Buchanan D.D., Cheng I.C., Cotterchio M., Figueiredo J.C., Gallinger S.J., Haile R.W., Jenkins M.A. (2020). Potential impact of family history–based screening guidelines on the detection of early-onset colorectal cancer. Cancer.

[121] Huyghe J.R., Bien S.A., Harrison T.A., Kang H.M., Chen S., Schmit S.L., Conti D.V., Qu C., Jeon J., Edlund C.K. (2019). Discovery of common and rare genetic risk variants for colorectal cancer. Nat. Genet..

[122] Gupta S., Provenzale D., Llor X., Halverson A.L., Grady W., Chung D.C., Haraldsdottir S., Markowitz A.J., Slavin T.P., Hampel H. (2019). NCCN Guidelines Insights: Genetic/Familial High-Risk Assessment: Colorectal, Version 2.2019. J. Natl. Compr. Canc. Netw..

